# Relationship between mental health and spiritual wellbeing among hemodialysis patients: a correlation study: Relação entre saúde mental e bem-estar espiritual em pacientes de hemodiálise: um estudo correlacional

**DOI:** 10.1590/1516-3180.2014.1321606

**Published:** 2014-02-01

**Authors:** Beatriz Bertolaccini Martínez, Rodrigo Pereira Custódio

**Affiliations:** I MD, MSc, PhD. Professor, Department of Medicine, Universidade do Vale do Sapucaí (Univás), Pouso Alegre, Minas Gerais, Brazil; II Nursing Student. Universidade do Vale do Sapucaí (Univás), Pouso Alegre, Minas Gerais, Brazil

**Keywords:** Spirituality, Mental health, Religion, Kidney failure, chronic, Dialysis, Espiritualidade, Saúde mental, Religião, Falência renal crônica, Diálise

## Abstract

**CONTEXT AND OBJECTIVE:**

The stress of living with a terminal disease has a negative impact on the mental health of hemodialysis (HD) patients. Spirituality is a potential coping mechanism for stressful experiences. Studies on the relationship between spirituality and mental health among HD patients are scarce. The purpose of this study was to evaluate the relationship between mental health and spiritual well-being among HD patients.

**DESIGN AND SETTING:**

Cross-sectional observational study on hemodialysis patients at a single center in Brazil, between January and December 2011.

**METHODS:**

: Mental health was assessed using the General Health Questionnaire and spiritual wellbeing was assessed using the Spiritual Wellbeing Scale; 150 HD patients participated in the study.

**RESULTS:**

: A significant correlation was found between mental health and spiritual wellbeing (P = 0.001). Spiritual wellbeing was the strongest predictor of mental health, psychological distress, sleep disturbance and psychosomatic complaints.

**CONCLUSION:**

Poor mental health was associated with lower spiritual wellbeing. This has important implications for delivery of palliative care to HD patients.

## Abstract (pt)

**CONTEXTO E OBJETIVO:**

O estresse de viver com uma doença terminal tem impacto negativo sobre a saúde mental de pacientes em hemodiálise. A espiritualidade é um mecanismo de enfrentamento em potencial para experiências estressantes. Estudos sobre a relação entre espiritualidade e saúde mental de pacientes em hemodiálise são escassos. O objetivo deste estudo foi avaliar a relação entre saúde mental e bem-estar espiritual dos pacientes em hemodiálise.

**TIPO DE ESTUDO E LOCAL:**

Estudo observacional e transversal de pacientes em tratamento de hemodiálise de centro único no Brasil, no período de janeiro a dezembro de 2011.

**MÉTODOS:**

A saúde mental foi avaliada pelo Questionário Geral de Saúde e o bem-estar espiritual foi avaliado usando a Escala de Bem-Estar Espiritual. Participaram do estudo 150 pacientes em hemodiálise.

**RESULTADOS:**

Foi encontrada correlação significante entre a saúde mental e o bem-estar espiritual (P = 0,001). Bem-estar espiritual foi o mais forte preditor de saúde mental, sofrimento psíquico, distúrbios do sono e queixas psicossomáticas.

**CONCLUSÃO:**

A saúde mental deficiente associou-se com menor bem-estar espiritual. Isso tem implicações importantes para a prestação de cuidados paliativos para pacientes em hemodiálise.

## INTRODUCTION

Psychiatric disorders are common among hemodialysis (HD) patients and are associated with increased morbidity and mortality, and reduced quality of life.^1^ Spirituality is an important factor in the quality of life of HD patients.^2^ According to Koenig et al.,^3^ spirituality is a personal quest to understand aspects of life, its meaning and the relationship with the sacred, which may or may not involve religious practices or formation of religious groups. Spirituality is a potential resource in relation to mental health and is a coping mechanism for stressful experiences.^4^

The relationship between spirituality and health is a relevant factor to be assessed among HD patients. However, there are few studies in the literature correlating spirituality and mental health in this population. 

## OBJECTIVE

This study was undertaken to evaluate the relationship between mental health and spiritual wellbeing among HD patients. 

## METHODS

This cross-sectional correlation study was approved by the Research Ethics Committee of Universidade do Vale do Sapucaн (Univбs), Brazil, and was conducted in accordance with the ethical standards of the 1964 Declaration of Helsinki and its subsequent amendments. Written informed consent was obtained from all patients prior to their inclusion in the study and anonymity was assured. 

One hundred and sixty-eight HD patients from a single medical center in Brazil were considered for the study, but were excluded if they had hearing impairment (three subjects), were younger than 18 years of age (three subjects), had been on hemodialysis for less than 12 months (nine subjects) or declined to participate in the study (two subjects). The remaining 151 patients were approached and asked to participate in the study. Of these, 150 agreed to participate and one subject declined, resulting in 150 patient completing the study. The recruitment period was from January to December 2011. 

Sociodemographic, economic and clinical data were obtained from all participants.

Mental health was assessed using the validated Brazilian Portuguese version of the General Health Questionnaire (GHQ).^5^ ^,^ ^6^ The GHQ measures mental health and consists of 60 items assessing the presence or absence of current non-psychotic symptoms and common mental disorders. The items are grouped into five subscales: psychological stress, death ideation, performance anxiety, sleep disturbance, and psychosomatic complaints. The GHQ also yields a total score, in which higher scores indicate lower mental health status.

The validated Brazilian Portuguese version of the Spiritual Wellbeing Scale (SWBS) was used to assess spiritual wellbeing.^7^ ^,^ ^8^ The SWBS contains 20 items, of which 10 assess religious wellbeing and 10 assess existential wellbeing. The total score, which is obtained by adding the scores for the two subscales, is a measurement of spiritual wellbeing.

Statistical analysis was carried out using the Statistical Package for the Social Sciences (SPSS) 18.0 (SPSS Inc., Chicago, IL, USA). The results were expressed as means ± standard deviations, medians and frequencies. Pearson's correlation coefficient (r) was used for bivariate analysis. Stepwise multiple logistic regression analysis was used to evaluate the correlation of mental health with other variables when P < 0.25 in the bivariate analysis. The significance level was set at 5% (P < 0.05).

## RESULTS

The sociodemographic, economic and clinical characteristics of the participants are listed in Table 1.

**Table 1:**
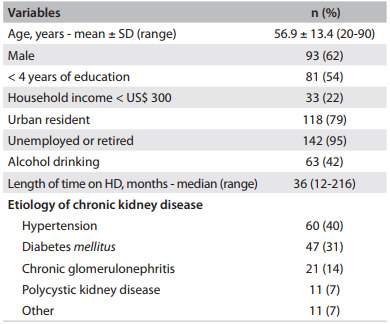
Characteristics of the hemodialysis patients (n = 150)

SD = standard deviation; HD = hemodialysis

The correlations of GHQ scores with the other variables are shown in Table 2. We found that high total GHQ scores (poor mental health) were associated with lower household income (P = 0.02); psychological stress was associated with younger age (P = 0.02) and with the female gender (P = 0.03); death ideation, with alcohol drinking (P = 0.03); high performance anxiety, with shorter length of time on HD (P = 0.005); and sleep disturbance, with longer length of time on HD (P = 0.01).

**Table 2:**
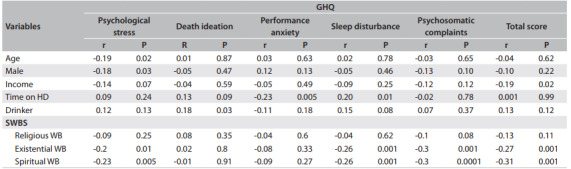
Correlation of General Health Questionnaire (GHQ) scores with Spiritual Wellbeing Scale (SWBS) scores and patient characteristics

HD = hemodialysis; WB = wellbeing: household income, alcohol drinking and existential and spiritual wellbeing

Poor mental health and the presence of psychological stress, sleep disturbance and psychosomatic complaints were associated with lower existential and spiritual wellbeing (P < 0.05). The multiple logistic regression results showed that spiritual wellbeing was the strongest predictor of mental health (P = 0.003), psychological stress (P = 0.006), sleep disturbance (P = 0.002) and psychosomatic complaints (P = 0.0003), as shown in Table 3.

**Table 3:**
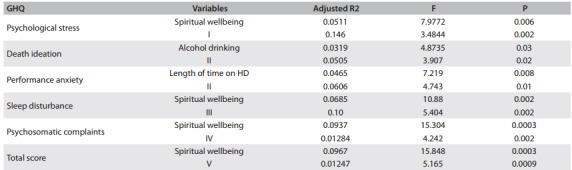
Logistic regression analysis on General Health Questionnaire (GHQ) scores, Spiritual Wellbeing Scale (SWBS) scores and patient characteristics

I = adjusted according to age, sex, household income, length of time on hemodialysis, alcohol drinking, existential wellbeing and spiritual wellbeing; II = adjusted according to length of time on hemodialysis and alcohol drinking; III = adjusted according to alcohol drinking, existential wellbeing and spiritual wellbeing; IV = adjusted according to sex, household income and existential, religious and spiritual wellbeing; V = adjusted according to household income, alcohol drinking and existential and spiritual wellbeing

## DISCUSSION

Recent advances in HD technology have increased the life expectancy of HD patients, but their quality of life has not changed appreciably. HD patients still experience a number of adverse situations relating to health, survival, limitations in activities of daily living, losses and biopsychosocial changes.^9^ These stressful situations result in psychiatric symptoms, especially depression and anxiety. Our results showed that there was an association between poor mental health and lower household income. This is consistent with the findings of studies conducted in the general population, indicating that financial stress may be an important source of distress in people's lives and may be associated with minor psychiatric disorders.^10^ ^,^ ^11^

The highest levels of psychological stress were observed among younger and female patients. Patients' life histories influence their present situation; thus, life experiences may trigger self-defense mechanisms against mental health disorders. In women, high levels of psychological stress may be attributed to factors including marriage, raising children, work and hormonal changes.^12^ ^-^ ^18^

Performance anxiety was associated with shorter length of time on HD. According to Diniz et al.,^19^ the initial phase of HD results in psychological distress due to the required use of life-support equipment, which limits patient autonomy. Some studies have reported that patients undergoing HD develop strategies over time for coping with the disease and treatment, thus resulting in less impact on their mental health.^20^ ^,^ ^21^

The strongest predictor of death ideation was alcohol abuse in our study. Studies have reported that alcohol abuse is associated with increased risk of suicidal behavior.^22^ ^,^ ^23^

The results revealed that poor mental health was associated with lower spiritual wellbeing, and that psychological stress, sleep disturbance and psychosomatic complaints were associated with lower existential and spiritual wellbeing. Spiritual wellbeing was a strong predictor of overall mental health, as well as psychological stress, sleep disturbance and psychosomatic complaints. This is in agreement with the findings of other studies, thus suggesting that spiritual wellbeing is a protective factor against minor psychiatric disorders.^8^ ^,^ ^24^

The higher the scores for spiritual wellbeing and, especially, for existential well-being are, the higher the likelihood of better mental health is. There are different ways of coping with disease and treatment. Suffering is a personal experience, but it is still possible to extract lessons from suffering and to rethink values, thereby giving life a new meaning.^25^

One limitation of this study is that the association between mental health and comorbidities, such as diabetes mellitus, bone disease, neurological deficits and cardiovascular diseases, was not investigated.

Further multicenter and prospective studies are necessary to better understand the relationship between spiritual wellbeing and mental health among HD patients. 

## CONCLUSION

In conclusion, our results revealed that spiritual wellbeing was negatively related to and the strongest predictor of psychological stress, sleep disturbance, psychosomatic complaints and mental health.
